# 

**Published:** 1907-10-19

**Authors:** 


### CONTENTS.

| | PAGE |
|---|---|
| **The British Pharmaceutical Codex** | 53 |
| **The Electrolytic Destruction of Bacilli** | 54 |
| **Annotations—** Morphinomania and Alcoholism—Proprietary Remedies in Australia—The Quarterly Journal of Medicine | 56 |
| **Medical Opinion and Movement** | 56 |
| **Hospital Clinics—** Syphilitic Arthritis. By Leonard Gamgee, F.R.C.S. | 57 |
| **Special Article—** What is Disease? What Diagnosis? | 60 |
| **Illustrative Cases—** Acute Bright's Disease Simulated by Renal Calculus | 62 |
| **Points in Surgery—** The Treatment of Fractures from a Common-sense Point of View | 63 |
| Microscopical Examination of Tissues during Operation | 64 |
| **Notes on Current Neurology—** Psychasthenia: its Evolution, Course, Diagnosis, and Treatment | 65 |
| **The Lighter Side of Medicine—** To a Club Patient | 66 |
| **Therapeutics—** Prescriptions and Dispensing | 67 |
| **Practical Notes on Diagnosis and Treatment** | 68 |
| **Ophthalmology—** Treatment of Foreign Bodies in the Cornea and their possible Sequelæ | 69 |
| **Practical Points** | 69 |
| **The Book World of Medicine and Science** | 70 |
| **Hospital Administration—** The Administration of Anæsthetics | 71 |
| The Melbourne Hospital | 72 |
| News and Coming Events | 73 |
| **Nursing Administration—** The Problem of Rural Midwifery | 74 |
| The Common Task | 75 |
| **Editor's Letter-Box—** The Hospital Saturday Fund | 76 |

